# Concurrent Medial Prefrontal Cortex and Dorsal Hippocampal Activity Is Required for Estradiol-Mediated Effects on Object Memory and Spatial Memory Consolidation

**DOI:** 10.1523/ENEURO.0271-19.2019

**Published:** 2019-08-19

**Authors:** Rosalind S.E. Carney

## Abstract

**Highlighted Research Paper:** Chemogenetic Suppression of Medial Prefrontal-Dorsal Hippocampal Interactions Prevents Estrogenic Enhancement of Memory Consolidation in Female Mice by, Jennifer J. Tuscher, Lisa R. Taxier, Jayson C. Schalk, Jacqueline M. Haertel, and Karyn M. Frick.

Steroid hormones such as estrogens and androgens affect the nervous system during *in utero* development and throughout adolescence, adulthood, and aging (Choleris et al., 2018). Steroid hormones have been associated with a prevalence in either men or women of certain neurologic disorders. For instance, Alzheimer’s disease and schizophrenia affect more women than men and vice versa, respectively (Irvine et al., 2012; Jackson et al., 2013). Studies have also shown that 17β-estradiol (E_2_), the predominant and most potent form of estrogen, can alter hippocampal neuroplasticity and therefore affect learning and memory. Exogenous E_2_ administration into the dorsal hippocampus (DH) of young female rodents enhances memory consolidation as shown by object recognition (OR) and object placement (OP; also known as object location) tasks (Tuscher et al., 2015). OR and OP are two object-based one-trial learning tasks that capitalize on rodents’ innate interest in novelty and relate to the “what” and “where” aspects of episodic memory consolidation and spatial memory, respectively (Frick et al., 2018a). DH E_2_ infusion immediately after the OR/OP training period restricts the effects of E_2_ to the time frame of memory consolidation (Tuscher et al., 2015). Post-training DH E_2_ infusion leads to an increase in spine density of CA1 pyramidal neurons during the time frame in which memory consolidation occurs (Gould et al., 1990; Woolley et al., 1990; Woolley and McEwen, 1993; MacLusky et al., 2005; Phan et al., 2012). Increased dendritic spine density in response to post-training E_2_ DH infusion was also observed in the medial prefrontal cortex (mPFC; Tuscher et al., 2016), a structure that expresses estrogen receptors and is also involved in memory consolidation. This observation prompted Tuscher and colleagues to examine in their *eNeuro* publication the effects of local infusion of E_2_ into the mPFC and the requirement for concurrent activity of the mPFC in E_2_-mediated memory consolidation in the DH.

Young female mice were ovariectomized (OVX) to eliminate the ovarian source of E_2_ that fluctuates during the estrous cycle, the rodent version of the menstrual cycle (Tuscher et al., 2015). Figure 1 shows a schematic illustration of the experimental approach. For OR training, a mouse was placed in a white square arena in which the upper left and right corners contained identical objects, one in each corner (Fig. 1*A*). The time spent exploring each object was recorded using tracking software, and mice remained in the arena until they had accumulated 30 s exploring the objects. If the mice remembered the identity of the training objects during testing 48 h later, then they should spend more time than chance (>15 s) exploring a novel object that had replaced one of the original training objects in the upper corners of the arena (Fig. 1*A*). The OP assessment differed from that of OR in two aspects: (1) OP testing occurred 24 h after training; and (2) whereas one of the training objects encountered during training remained in its original location during testing, the second training object had been moved to a lower corner of the arena (Fig. 1*B*). Spending more time than chance with the moved object indicated that spatial memory was intact.

**Figure 1.**
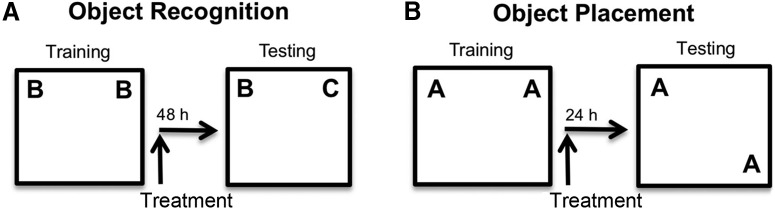
Schematic illustration of OR and OP behavioral assessments. ***A***, Treatment administered after training reveals treatment effect on memory consolidation of a novel object versus a familiar object. ***B***, Treatment administered after training reveals treatment effect on memory consolidation of the placement of a previously encountered object in a new location versus original placement of another previously encountered object (Adapted from Figure 1 in Tuscher et al., 2019).

OVX mice were implanted with bilateral cannulae targeted to the mPFC one week before training in the OR and OP tasks, which were performed in separate groups of mice. Immediately after training, mice received mPFC infusions of either cyclodextrin-encapsulated E_2_ dissolved in sterile saline or the vehicle control, 2-hydroxypropyl-β-cyclodextrin (HBC) dissolved to the same concentration of cyclodextrin as the E_2_-containing solution. In both the OR and OP behavioral assessments, E_2_-infused mice spent more time exploring the novel or moved objects than would be expected by chance alone and significantly more time compared to vehicle-infused mice (Fig. 2). These observations showed that local effects of E_2_ within the mPFC replicate the enhancement of memory consolidation and spatial memory that resulted from local infusion of E_2_ within the DH.

**Figure 2.**
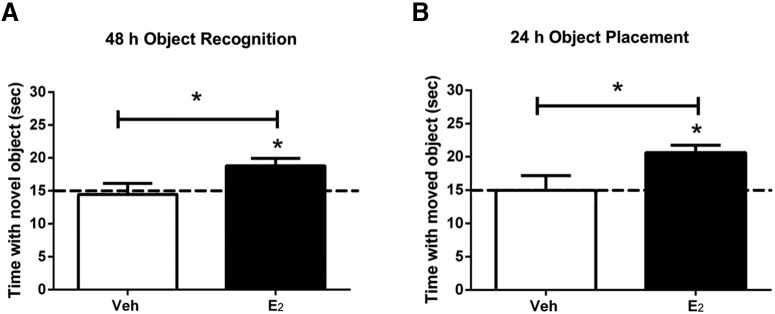
Infusion of E_2_ into the mPFC immediately after training enhances memory consolidation. Mice infused with E_2_ directly into the mPFC spent significantly more time than chance (dashed line at 15 s) with the novel object (***A***) when tested 48 h after training or with the moved object (***B***) 24 h after training. Mice infused with vehicle (Veh) into the mPFC did not spend more time than chance with the novel (***A***) or moved (***B***) objects. These data suggest that E_2_ can improve the consolidation of object memories by acting directly within the mPFC. Bars represent the mean ± SEM; *p < 0.05 relative to chance and the vehicle group (Adapted from Figure 1 in Tuscher et al., 2019).

Although a causal link between E_2_-mediated spinogenesis and memory consolidation has not yet been established (Sheppard et al., 2019), signaling mechanisms are common to both processes (Frick et al., 2018a). Therefore, Tuscher and colleagues examined local E_2_-mediated effects on spinogenesis in the mPFC. Two weeks after the completion of the OR and OP behavioral assessments, mice received an infusion of E_2_ or vehicle control into the mPFC; 2 h later the brains of the mice were harvested and processed for Golgi staining. Dendritic spine analysis was performed in regions in which increased E_2_-mediated spinogenesis was previously observed, namely, Layer II/III of the mPFC and CA1 of the DH. Spine density was increased apically but not basically when E_2_ was locally infused into the mPFC, but spine density in the DH was unchanged. It is not known why local infusion of E_2_ did not affect spinogenesis in the DH; perhaps the time frame was insufficient to detect any alterations or E_2_ infusion directly into the DH has more hierarchical, rather than reciprocal, effects on another brain region.

The observations described thus far provided evidence that E_2_ acting locally in the mPFC enhances memory consolidation and increases local spinogenesis. Next, the authors sought to determine if concurrent activation of the mPFC is required for enhanced E_2_-mediated, DH-dependent memory consolidation. To do so, they employed a designer receptors exclusively activated by designer drugs (DREADD) approach to suppress activity in the mPFC while simultaneously infusing E_2_ into the DH. Immediately after ovariectomy, viral DREADD constructs or saline (sham condition) were infused bilaterally into the mPFC, and cannulae were also placed bilaterally in the DH for future infusions. The AAV-CaMKIIα-eGFP construct induced expression of green fluorescent protein (GFP) in excitatory neurons in the mPFC three weeks later, at the time when behavioral assessments were initiated. At the time of the surgical procedure, another group of OVX mice received bilateral mPFC injections of an AAV-CaMKIIα-HA-hM4Di-IRES-mCitrine viral construct. Binding of the clozapine-N-oxide (CNO) ligand to the hM4Di DREADD suppresses the activity of the excitatory CaMKIIα-expressing cells, thereby inhibiting the mPFC. After training in the OR or OP tasks, mice received DH infusion of E_2_ or vehicle control at the same time that CNO was administered systemically to all experimental groups, at a concentration of CNO that does not impair memory consolidation on its own (Tuscher et al., 2018). In both the OR and OP tasks, memory consolidation was enhanced in mice in which E_2_ was infused directly into the DH but in which mPFC activity was not suppressed (sham + E_2_, GFP + E_2_ groups; Fig. 3) compared to experimental groups that received vehicle control (sham + HBC, GFP + HBC, hM4Di + HBC; Fig. 3). Suppression of excitatory signaling in the mPFC blocked the E_2_-mediated DH-dependent enhancement of memory consolidation (hM4Di + HBC; Fig. 3) showing, for the first time, that mPFC activity is required for memory consolidation when E_2_ is locally infused into the DH.

**Figure 3.**
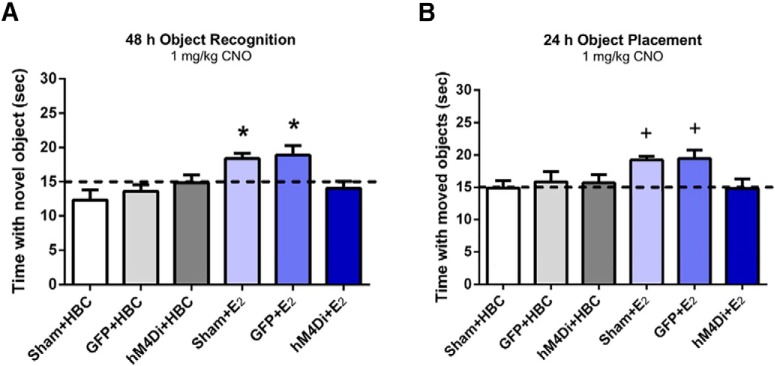
Chemogenetic suppression of the mPFC immediately after training prevents the memory enhancement induced by DH E_2_ infusion. Sham, GFP, or hM4Di control groups receiving CNO + Veh did not spend significantly more time than chance (15 s) with the novel object (***A***) when tested 48 h after training or with the moved object (***B***) 24 h after training. In contrast, sham or GFP mice receiving CNO + E_2_ immediately after training did spend significantly more time than chance with the novel (***A***) and moved (***B***) and objects, displaying intact OR and OP memory. However, hM4Di mice treated with CNO+E_2_ immediately after training did not demonstrate intact memory, suggesting that DREADD-mediated suppression of the mPFC blocks the beneficial mnemonic effects of DH-infused E_2_. Bars represent the mean ± SEM; *p < 0.05 relative to chance, all Veh-infused groups, and the hM4Di-E_2_ group; +*p* < 0.05 relative to chance and the sham + Veh-infused and hM4Di-E_2_ groups (Adapted from Figure 4 in Tuscher et al., 2019).

The *eNeuro* publication by Tuscher and colleagues highlights the complexity of E_2_-mediated memory consolidation processes and raises several new avenues of research. The finding that local E_2_ infusion into the mPFC affects a cognitive process that is heavily associated with the hippocampus underscores the importance of research into other brain structures involved in cognition that express steroid receptors. The mPFC and DH interaction could be regulated by direct or indirect neural circuitry. It will also be interesting to determine if local effects of E_2_ within the mPFC involve classic, genomic signaling via estrogen receptors ERα and ERß or the rapid signaling that results from extracellular ligand binding to the G protein-coupled receptor GPER. Coupling the use of estrogen agonists and antagonists in the DH with chemogenetic suppression of other brain regions will help resolve the interactions between signaling mechanisms and neural circuitry. Such receptor manipulation could also reveal the relevance of local estradiol production within the hippocampus that is unaffected by ovariectomy.

Tuscher and colleagues’ findings in this *eNeuro* publication may also help resolve discrepancies in the literature regarding the effects of E_2_ on cognition by emphasizing the need to examine other brain regions involved in memory consolidation. But more complexities exist, such as the effects of steroid signaling in young versus older or female versus male rodent models (Frick et al., 2018b). It was expected that hormone replacement therapy in postmenopausal women would have a protective effect against cognitive dysfunction. However, some of these studies used conjugated equine estrogens, rather than E_2_, which may explain the lack of beneficial effects (Luine, 2014). Moreover, it has recently been shown in a mouse model of Alzheimer’s disease that E_2_ fluctuations during the ovarian cycle have adverse effects on cognition (Broestl et al., 2018). Therefore, a plethora of age, sex, and brain region considerations may be needed to fully elucidate new targets for drug development that address steroid-related effects on cognitive functions, such as memory consolidation.
